# Efficient Production of a Bioactive Bevacizumab Monoclonal Antibody Using the 2A Self-cleavage Peptide in Transgenic Rice Callus

**DOI:** 10.3389/fpls.2016.01156

**Published:** 2016-08-09

**Authors:** Lei Chen, Xiaoyu Yang, Da Luo, Weichang Yu

**Affiliations:** ^1^School of Life Sciences, Sun Yat-sen UniversityGuangzhou, China; ^2^Shenzhen Research Institute, The Chinese University of Hong KongShenzhen, China; ^3^College of Life Sciences, Shenzhen UniversityShenzhen, China

**Keywords:** Bevacizumab, monoclonal antibody (mAb), plant transformation, 2A self-cleavage peptide, plant made antibody, rice

## Abstract

Bevacizumab, a humanized monoclonal antibody (mAb) targeting to the vascular endothelial growth factor (VEGF), has been widely used in clinical practice for the treatment of multiple cancers. Bevacizumab was mostly produced by the mammalian cell expression system. We here reported the first plant-derived Bevacizumab by using transgenic rice callus as an alternative gene expression system. Codon-optimized Bevacizumab light chain (BLC) and Bevacizumab heavy chain (BHC) genes were designed, synthesized as a polyprotein with a 2A self-cleavage linker peptide from the *Foot-and-mouth disease virus*, cloned into a plant binary vector under a constitutive maize ubiquitin promoter, and transformed into rice nuclear genome through *Agrobacterium*-mediated transformation. Southern blot and western blot analyses confirmed the integration and expression of BLC and BHC genes in transgenic rice callus. Enzyme-linked immunosorbent assay (ELISA) analysis indicated that the rice-derived Bevacizumab mAb was biologically active and the recombinant mAb was expressed at high levels (160.7–242.8 mg/Kg) in transgenic rice callus. The mAb was purified by using protein A affinity chromatography and the purified antibody was tested for its binding affinity with its target human VEGF (hVEGF) antigen by ELISA. Rice callus produced Bevacizumab and a commercial Bevacizumab (Avastin) were shown to have similar binding affinity to hVEGF. These results indicated that rice callus produced Bevacizumab could have similar biological activity and might potentially be used as a cost-effective biosimilar molecule in future cancer treatment.

## Introduction

Plants including the whole body, specific tissues or organs and plant-derived suspension cells are considered as attractive and competitive platforms for recombinant protein production. Compared with *Escherichia coli* and mammalian cells, plant-based production systems have been demonstrated to have many advantages. First, plant-based production systems are more flexible, as well as easier to operate, manage, and scale up, and thus can decrease the production cost of recombinant proteins to a great extent. Second, plants, similar to mammalian cells, are able to perform post-translational modification of recombinant proteins such as glycosylation, and thus additional cost for *in vitro* protein modifications can be avoided (Frenzel et al., 2013; Sil and Jha, 2014; Kolotilin et al., 2015). Third, long-term continuous recombinant protein production can be realized in plant platforms because transgenes can be stably integrated into the nuclear genome of host plants, faithfully inherited, and expressed in later generations. Furthermore, plant-derived recombinant proteins may be safer than those from *E. coli* or mammalian cells because the risk of contamination with human pathogens, which is always a concern when using mammalian cells as a bioreactor, can be well circumvented by plant-based production systems (Thie et al., 2008; Ni and Chen, 2009; Merlin et al., 2014). Because of these properties, various bioactive pharmaceutical proteins have been produced in plants since first expression of a human growth hormone in transgenic tobacco and sunflower callus tissue (Barta et al., 1986), and expression of antibodies, vaccines, hormones, growth factors, and cytokines (De Muynck et al., 2010; Desai et al., 2010; Xu et al., 2011; Huang and McDonald, 2012).

Monoclonal antibodies (mAbs) are protein complexes containing four subunits with two identical light chains (LC) and two identical heavy chains (HC). MAbs are important in biological research, clinical diagnosis, and recently immunotherapy for various diseases and cancer (De Muynck et al., 2010). Unlike other single chain recombinant proteins, the production of mAbs requires simultaneous expression of two genes coding for both LC and HC, and the correct folding of four polypeptides linked by disulphide bonds. The discovery that plant can efficiently express and correctly assemble functional antibodies (Hiatt et al., 1989) have made plants an alternative antibody production system, and since then, many recombinant antibodies have been produced in various plants, including moss (Decker and Reski, 2008), algae (Franklin and Mayfield, 2005), and higher plants (Stoger et al., 2005; De Muynck et al., 2010; Xu et al., 2011, 2012; Schillberg et al., 2013).

Previously, the LC and HC genes of a mAb were expressed in two different expression cassettes on one T-DNA region of a vector, or expressed separately in individual vectors which were co-transformed to the same plant, or expressed in different transgenic plants which were cross-fertilized to produce the functional antibody (De Muynck et al., 2010; Ko, 2014). Because the two genes are expressed separately, it is difficult to control their relative expression level even though identical regulatory elements are used. In fact, most of the previous reports have produced unbalanced LC and HC in both transgenic plants and mammalian cells (Voss et al., 1995; Law et al., 2006; De Muynck et al., 2010; Chng et al., 2015). A varied LC:HC ratio is usually unfavorable for the folding of functional mAbs, and affects both the level and quality of mAbs (Schlatter et al., 2005; Law et al., 2006; Lee et al., 2009; Ho et al., 2013b). The use of internal ribosome entry site (IRES) to translate two polypeptides (LC and HC) from one mRNA also results in an unbalanced expression because of the lower efficiency of the IRES directed downstream gene expression by a cap-independent translation mechanism (Hennecke et al., 2001; Ho et al., 2012, 2013a,b). In contrast, the use of 2A peptide from the Aphthovirus *Foot-and-mouth disease virus* (FMDV) for high level mAb expression has been reported in both the human embryonic kidney 293 and the Chinese hamster ovary (CHO) cells (Fang et al., 2005; Chng et al., 2015), but this strategy for mAb expression in transgenic plant system has not been reported so far.

Bevacizumab is a humanized mAb that targets to the vascular endothelial growth factor (VEGF) antigen (Presta et al., 1997; Ferrara et al., 2005), which is widely over expressed in a variety of human solid tumors and plays a key role in tumor angiogenesis (Ellis and Hicklin, 2008; Goel and Mercurio, 2013; Domigan et al., 2015). Bevacizumab neutralizes VEGFs, prevents their interactions with VEGFR-1 and VEGFR-2 receptors, and thus blocks the downstream signal transductions for tumor angiogenesis (Wang et al., 2004). Bevacizumab is derived from the murine VEGF mAb A4.6.1. It has 93% human and 7% murine sequence, and has similar biochemical and pharmacologic properties to the original murine mAb. It neutralizes all isoforms of human VEGF (hVEGF) with high affinity and inhibits VEGF-induced proliferation of endothelial cells and tumor angiogenesis, but with reduced immunogenicity and longer circular half-life as compared to the murine antibody (Gerber and Ferrara, 2005). It has been widely applied in clinical practice of metastatic colorectal cancer, glioblastoma, non-small-cell lung cancer, metastatic kidney cancer, advanced cervical cancer, platinum-resistant ovarian cancer (Giantonio, 2006; Ali et al., 2008; Tonini et al., 2013; Tewari et al., 2014; Liu and Matulonis, 2015; Mason, 2015)^1^. Bevacizumab (Avastin) is approved by the US Food and Drug Administration for clinical use in cancer therapy (Bellmunt, 2007; Pal et al., 2014).

Although increasing evidence suggests the great potency of Bevacizumab for various disease therapies, there are still some obstacles which limit its widespread applications (Hayashi et al., 2014). One of the shortcomings is the high cost of Bevacizumab-mediated therapy. The high cost of Bevacizumab is greatly influenced by its production, which is solely dependent on mammalian expression systems (Makino et al., 2011; Spadiut et al., 2013).

In this study, we reported the first plant made Bevacizumab by using transgenic rice callus as a gene expression system. Rice is both a main stuff crop and a model plant for biological studies because of its small genome and the high efficient gene transformation system. Rice seeds and suspension cultures have been reported as good systems for the production of many high value bioactive proteins (Kim et al., 2008; He et al., 2011; Kuo et al., 2013; Corbin et al., 2016; Fujiwara et al., 2016). Codon-optimized Bevacizumab LC and HC genes were designed, synthesized as a polyprotein with a 2A self-cleavage linker peptide from the FMDV, cloned into a plant binary vector under a constitutive maize ubiquitin promoter, and transformed into rice nuclear genome through *Agrobacterium*-mediated transformation. High levels of biologically functional Bevacizumab were expressed and purified by affinity chromatography. An enzyme-linked immunosorbent assay (ELISA) binding assay showed that the rice expressed Bevacizumab and the commercial Avastin had similar binding affinity to the VEGF target.

## Materials and Methods

### Plant Materials

Rice (*Oryza sativa* var. japonica cv. Nipponbare) was used in this study. Dehusk mature healthy seeds were washed with Milli-Q water followed by 70% (v/v) ethanol for 30 s and then immersed into 1.5% sodium hypochlorite for 30 min with vigorous shaking. After being washed with sterilized Milli-Q water three times, the seeds were placed on N6 medium (Chu et al., 1975) supplemented with 2 mg/L 2,4-dichlorophenoxyacetic acid (N6D medium) at 28°C in the darkness for 3 weeks in order to induce callus from mature embryo. The obtained calli were subcultured every 3 weeks and prepared for subsequent *Agrobacterium*-mediated transformation.

### Construction of Plant Binary Vector

**FIGURE 1:**
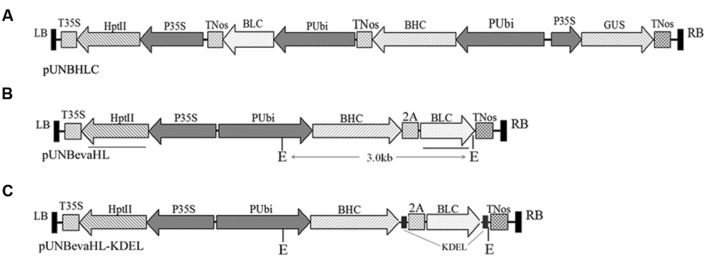
**T-DNA regions of constructs for rice transformation.** **(A)** pUNBHLC construct with Bevacizumab LC (BLC) and HC (BHC) genes expressed in two expression cassette; **(B)** pUNBevaHL construct with BLC and BHC expressed as a polyprotein linked with a FMDV 2A self-cleavage peptide; **(C)** pUNBevaHL-KDEL construct with BLC and BHC expressed as a polyprotein as in **(B)**, but a KDEL ER retention peptide sequence was added to the C-termini of the coding regions of both BLC and BHC genes. Lysogeny broth (LB) and RB denote the left and right borders of the T-DNA region of the binary vector. TNos and T35S are terminators of the *Agrobacterium* nopaline synthase gene and the CaMV 35S expression cassette, respectively. 2A is the self-cleavage peptide from foot-and-mouth disease virus, and KDEL is the ER retention signal peptide. Two *Eco*RI (E) restriction enzyme digestion sites are indicated on pUNBevaHL and pUNBevaHL-KDEL constructs in **(B**,**C)**, and the ∼3 kb fragments flanked by the two *Eco*RI sites are indicated. The bars under pUNBevaHL construct **(B)** show the positions of HptII and BLC probes used in Southern blot analysis.

The LC and HC peptide sequences of Bevacizumab were obtained from DrugBank database^2^. Genes coding for the Bevacizumab light chain (BLC) and Bevacizumab heavy chain (BHC; GenBank, KX119516 and KX119517) were optimized by an online codon optimizer software^3^ using the characteristics of rice codon usage bias^4^, based on the amino acid sequences of Bevacizumab (**Figures 1A,B**). An endoplasmic reticulum (ER) retention signal peptide (KDEL) was added to the C-termini of both BLC and BHC for targeted expression of the recombinant proteins to ER (**Figure 1C**).

To make a polyprotein of the BLC and BHC, a 2A sequence (QLLNFDLLKLAGDVESNPGP) from FMDV (Donnelly et al., 2001) was inserted between the BHC and BLC (**Figures 1B,C**). A GSG peptide linker was inserted immediately preceding the 2A sequence to improve the cleavage efficiency (Chng et al., 2015).

All the coding sequences were synthesized and placed under a maize ubiquitin promoter and cloned into a plant binary vector pUN1390 with *Pac*I and *Mlu*I to make pUNBevaHL and pUNBevaHL-KDEL (**Figures 1B,C**). pUNBevaHL-KDEL differed from pUNBevaHL in the presence of the ER retention sequences on both BLC and BHC chains.

### Plant Transformation

*Agrobacterium*-mediated transformation was performed based on the method of Nishimura et al. (2006). Recombinant binary vectors were introduced into the *Agrobacterium* strain EHA105 by freeze–thaw method (Weigel and Glazebrook, 2006). The resulted Agrobacteria were cultured in lysogeny broth (LB) liquid medium supplemented with 50 mg/L kanamycin and 50 mg/L rifampicin at 28°C with 220 rpm of agitation overnight. These cultures were centrifuged and re-suspended in AAM medium (Nishimura et al., 2006) containing 100 μM acetosyringone, to make OD600 of the *Agrobacterium* suspension in the range of 0.05–0.10 for rice transformation. The newly induced rice calli were dried with sterilized filter paper, kept in *Agrobacterium* culture for 15 min with short agitation, dried again with sterilized filter paper and finally placed on co-cultivation medium at 28°C in darkness for 3 days. The resulted calli were then transferred onto selection medium (N6D medium supplemented with 500 mg/L cefotaxime and 50 mg/L hygromycin B) and incubated at 28°C in darkness until the appearance of hygromycin B-resistant calli. After three rounds of 3-week subculture on selection medium, the hygromycin B-resistant calli were confirmed by southern blot analysis (**Supplementary Method S1**), and regenerated according to the method of Nishimura et al. (2006).

### Western Blot

Total protein was extracted from calli of wild type and selected transgenic rice lines. Calli were ground with a mortar and pestle in liquid nitrogen. The resulting fine powder was added to an equal volume of extraction buffer (200 mM Tris–HCl, pH 8.0, 100 mM NaCl, 400 mM sucrose, 10 mM ethylenediaminetetraacetic acid [EDTA], 1 mM phenylmethylsulfonyl fluoride, 0.05% Tween 20), mixed gently, and incubated on ice for 10 min. Samples were centrifuged twice at 13,000 rpm for 10 min at 4°C. Then 25 μg of protein extracts were mixed with sodium dodecyl sulfate (SDS) loading buffer, separated on a 10% SDS polyacrylamide gel electrophoresis (PAGE) under reducing (boiled for 5 min with 5% 2-mercaptoethanol) or non-reducing conditions, and immunoblotted onto a polyvinylidene difluoride membrane (Millipore Corporation, USA). The membrane were blocked for 2 h at room temperature with 3% (w/v) bovine serum albumin (BSA) in 1× phosphate-buffered saline (PBS) containing 0.1% (v/v) Tween-20 (PBST, 137 mM NaCl, 2.7 mM KCl, 8.1 mM Na_2_HPO_3_, 1.5 mM KH_2_PO_3_, pH7.5) and probed for 2 h at room temperature with peroxidase-conjugated affinipure goat anti-human IgG (LC+HC) antibody (ProteinTech Group, USA) diluted in blocking buffer [1× PBST with 3% (w/v) BSA] at a ratio of 1:5000. The blotted membrane was washed three times with 1× PBST and signals were developed by using a FUJI Medical X-ray film (Fujifilm, Japan). A commercial Bevacizumab mAb from Roche (Avastin, Lot No: H0129; Roche Pharma, Switzerland) was used as a positive control.

Assuming that there is a range in which Western blot image intensity is linearly proportional to protein concentration, the relative expression of BLC and BHC can be determined by analyzing the image intensity. Western blot images were analyzed by an ImageJ program^5^ to determine the density of the BLC and BHC bands of each transgenic line. The BLC:BHC ratio was calculated by dividing the density of BLC by that of BHC.

### Biological Activity of Rice-Derived Bevacizumab

Biological activities of the rice-derived Bevacizumab from transgenic rice callus were detected using an ELISA Kit (SHIKARI Q-BEVA, Matriks Biotechnology Co. Ltd., Turkey). The standards and samples were diluted at 1:100 using assay buffer supplied in the kit, and were incubated in pre-coated microtiter plate with hVEGF at 25°C for 1 h. After being washed three times with 1× PBST, a biotin labeled hVEGF was added to the microtiter plate and incubated at 25°C for 1 h. Following 1 h incubation, the wells were washed three times with 1× PBST, and then a streptavidin conjugated horseradish peroxidase (HRP) was added and incubated at 25°C for 0.5 h. Then, the wells were washed three times with 1× PBST and the bound enzymatic activity was detected by adding a TMB (3,3′,5,5′-tetramethylbenzidine) substrate solution. After incubation at 25°C for 20 min, the substrate reaction was stopped by adding a TMB stop solution into each well and the plates were read at a wavelength of 450 nm in a microplate reader (BioTek, USA).

### Quantification of Rice-Derived Bevacizumab

A standard curve was produced by using the commercial Bevacizumab at concentrations of 0, 3, 10, 30, and 100 μg/mL. The mAb concentration in mg/Kg fresh weight (FW) in each transgenic rice sample was calculated by comparing the OD450 reading to the prepared Bevacizumab standard curve. Three replicates were measured for each sample and the data were statistically analyzed.

### Antibody Purification

Total soluble proteins were extracted from 50 g calli of two transgenic lines (UNBevaHL-3-15 and UNBevaHL-KDEL-5) as described before. Extracted proteins were filtered through 0.45 μm filters and loaded onto HiTrap Protein A HP columns (GE Healthcare) pre-equilibrated with the binding buffer (0.02 M sodium phosphate buffer, pH 7.0). Five column volumes of binding buffer were used to wash the columns, and the mAbs bound to protein A were eluted with the elution buffer (0.1 M Glycine-HCl, pH 2.7), collected into tubes containing one-fifth volume of 1 M Tris–HCl (pH 9.0) to bring to pH 7.0. The obtained mAbs were concentrated by ultrafiltration (molecular weight cut-off, 30 kDa, Millipore, USA), quantified by ELISA as described before, and then used for analyses of binding activities and glycosylation. The purity of mAbs was analyzed by running in a non-reducing SDS-PAGE followed by a Coomassie blue staining.

### VEGF Binding Affinity Assay

The binding affinity of purified rice-derived Bevacizumab to its antigen was assessed by using a VEGF antigen binding ELISA. The antigen hVEGF (11066-HNAH, Sino Biological Inc, Beijing, China) solution in 1× PBS (50 μL, 0.5 μg/mL) was coated onto 96-well microtiter plates (Nunc^®^Model No. 4-42404, Roskilde, Denmark) overnight at 4°C. The wells were washed five times with 200 μL of 1× PBST between each step to completely remove unbound material. The wells were blocked with 200 μL 1% BSA (w/v) in 1× PBS at 25°C for 2 h. The purified mAbs were serially diluted to concentrations of 0.0041–9.0 μg/mL in 1× PBS. One hundred microliters of each diluted solution was loaded into the wells, and incubated for 2 h at 25°C. A commercial Bevacizumab was used as a reference, 1× PBS buffer was used as the negative control. After washing, bound antibodies to VEGF were incubated with 50 μL 1% BSA in 1× PBS containing 1 μg/mL goat-derived HRP-conjugated anti-human IgG (HC+LC) antibody (ProteinTech Group, USA). One hundred microliters of TMB substrate solution was added to each well, and the reaction was stopped after 20 min incubation at 25°C. The absorbance at 450 nm was read using a Microplate Reader (BioTek, USA). All samples were assayed in triplicates.

### N-glycan Analysis

The rice-derived Bevacizumab was purified as described previously, digested with trypsin, and extracted from gel pieces according to Kolarich et al. (2006). The subsequent fractionation of the peptides by capillary reversed-phase chromatography and detection by a quadrupole time-of-flight Ultima Global (Waters) mass spectrometer were performed according to Van Droogenbroeck et al. (2007). The mass spectrometer data of tryptic peptides were analyzed against the *in silico*-generated tryptic digestion of the Bevacizumab amino acid sequence using the Peptide Mass program^6^. Based on the tryptic peptide data sets, tryptic glycopeptide data sets were generated by the addition of glycan mass increments to the masses of the identified glycopeptides.

### Statistical Analysis

ELISA data were subjected to one-way analyses of variance with OriginPro8 (Version8E, Origin Lab Corporation, MA, USA) and presented as means ± standard error of three replicates.

## Results

### Construction and Expression of Bevacizumab mAb in Transgenic Rice

To efficiently produce the recombinant mAb, we designed two strategies for the *BLC* and *BHC* gene expression. In one design, the *BLC* and *BHC* genes were expressed separately in two gene expression cassettes both driven by the maize ubiquitin promoter (**Figure 1A**). In the other design, the *BLC* and *BHC* were expressed as a polyprotein gene linked by a FMDV 2A self-cleavage peptide (Donnelly et al., 2001), which can be self-processed to release the BLC and BHC proteins (**Figure 1B**). To avoid plant-specific glycosylation in expressed proteins, an ER retention signal (KDEL) was added to the C-termini of both the BLC and BHC proteins (**Figure 1C**).

The three constructs were introduced into rice nuclear genome by *Agrobacterium*-mediated transformation. Transgenic calli were selected on medium supplemented with hygromycin B based on the expression of the *Hpt*II selection marker gene. Stable transgenic lines were confirmed by Southern blot analysis using full-length *BLC* and *hpt*II as probes. Fourteen transgenic events were confirmed and the copy numbers of each transgenic line were revealed. Among them, four transgenic lines had a single copy transgene, six lines had two copies, and the rest of lines had three or more copies (**Supplementary Figure S1**). Transgenic calli were regenerated to produce transgenic rice plants which showed normal phenotypes similar to the wild type rice (**Supplementary Figure S2**).

**FIGURE 2:**
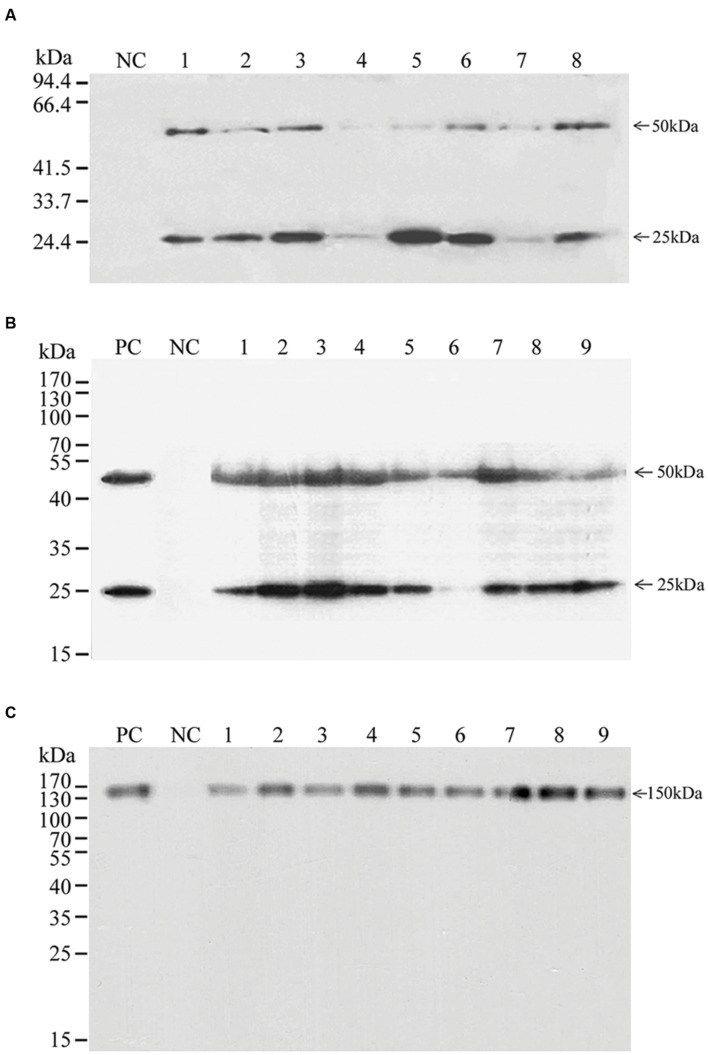
**Western blot of the expression of BHC, BLC, and Bevacizumab mAb in transgenic rice callus.** The BLC, BHC, and Bevacizumab mAb were detected with a goat anti-human IgG (LC and HC) antibody under reducing **(A,B)** and non-reducing conditions **(C)**. **(A)** BHC and BLC were expressed from different gene expression cassette, **(B,C)** BHC and BLC were expressed as a polyprotein separated with the 2A peptide. PC is a positive control with a commercial Bevacizumab mAb; NC is a negative control with the total soluble proteins from non-transformed callus; lanes 1–8 in **(A)**, 1–5 and 6–9 in **(B,C)** are total soluble proteins from different transgenic events transformed with pUNBHLC, pUNBevaHL, and pUNBevaHL-KDEL, respectively. Protein molecular weights are indicated on the left side of each panel. BHC (50 kDa), BLC (25 kDa), and mAb (150 kDa) are indicated on the right side of each panel.

Selected transgenic rice callus lines were further tested by SDS-PAGE analysis to confirm the expression of the transgene at the protein level (**Figure 2**). All tested transgenic lines produced both the BLC and BHC proteins (**Figures 2A,B**). Transgenic lines with transgenes expressed in two expression cassettes (**Figure 2A**) produced variable levels of BLC (∼25 kDa) and BHC (∼50 kDa), and in most lines signals of expressed BLC were stronger than the BHC. The ratios of BLC to BHC in different transgenic lines were from 0.95 to 19.2 with an average of 4.32 (**Supplementary Table S1**), indicating unbalanced expression of the BLC and BHC proteins. Thus, further analysis of transgenic lines with this construct was not performed.

In contrast, BLC and BHC in most transgenic lines with the 2A peptide constructs were proportional, although variations in the expression levels were observed among different transgenic events. The BLC:BHC ratios from different transgenic lines were from 0.11 to 1.90 with an average of 0.99, similar to the ratio of the commercial Bevacizumab (Avastin) sample (0.99) (**Supplementary Table S1**), indicating that about equal molecules of BLC and BHC peptides were produced by the self-cleavage of 2A peptide linker (**Figure 2B**). In all transgenic lines, there were only two major bands of the ∼25 kDa BLC and the ∼50 kDa BHC, indicating that he self-cleavage of the 2A peptide was complete and the majority of expressed protein was intact with little degradation.

To find out whether the expressed BLC and BHC had assembled correctly into mAb tetramers, a non-reducing SDS-PAGE that would not disrupt disulphide bonds of the antibody was performed (**Figure 2C**). Five transgenic lines transformed with the pUNBevaHL and four lines with the pUNBevaHL-KDEL expressed comparable levels of mAb with a molecular weight of about 150 kDa, similar to the positive control of the commercial Bevacizumab. In all transgenic lines, there was only one ∼150 kDa band of the mAb, indicating that there was no protein degradation.

### Biological Activity and Quantification of Rice-derived Bevacizumab mAb

**FIGURE 3:**
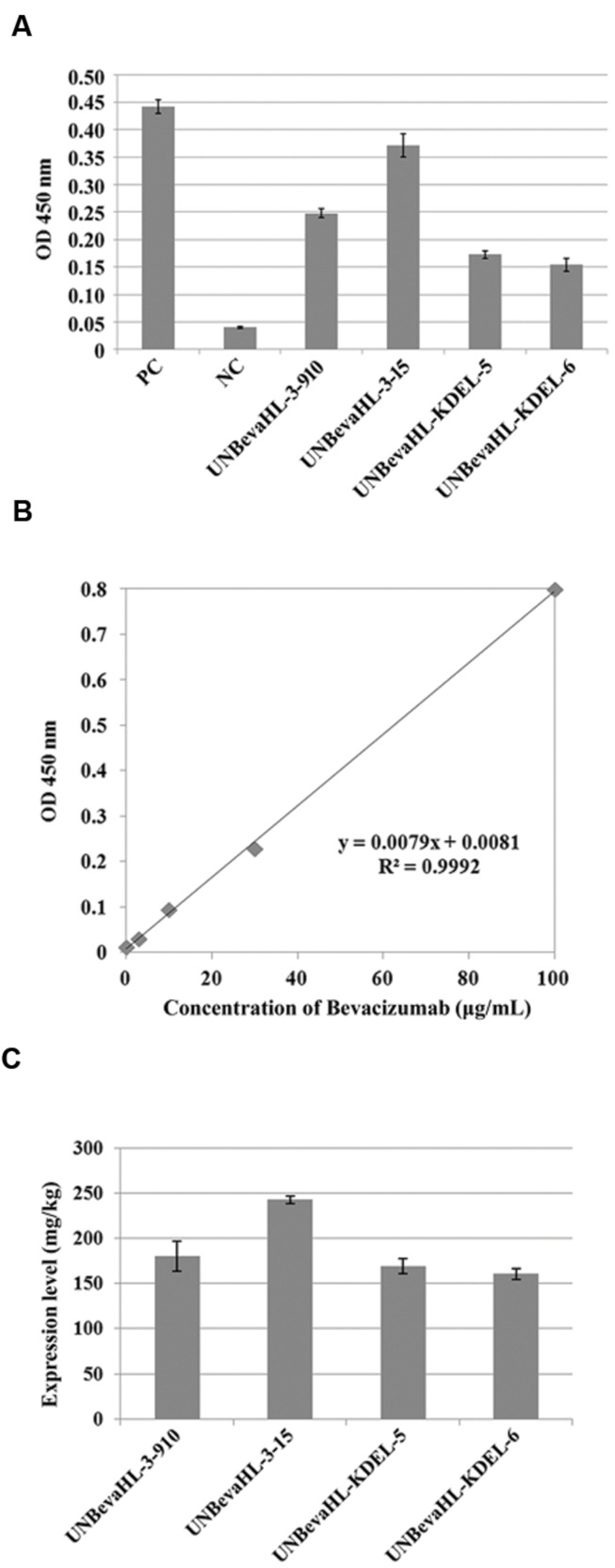
**Functional analysis of plant expressed Bevacizumab mAb and qualification of the expression levels in transgenic plants by ELISA.** **(A)** The binding activity of rice-derived Bevacizumab to hVEGF antigen was determined by ELISA with a positive control of the commercial Bevacizumab mAb and a negative control of non-transformed plants. Two transgenic lines each from pUNBevaHL and pUNBevaHL-KDEL transformed plants were analyzed in triplicates. **(B)** Standard curve of Bevacizumab. **(C)** mAb expression levels in transgenic plants. The experiment was performed in triplicates, and the means and errors of mAb contents in different transgenic plants were presented in graph.

The finding that transgenic rice callus could produce correctly assembled mAb tetramers indicated that this plant expressed mAb could be functional. Biological activity of rice-derived Bevacizumab was detected by ELISA, an assay based on the highly specific antigen–antibody interaction. The interactions of rice-expressed Bevacizumab to hVEGF were confirmed in all four tested transgenic lines, similar to the positive control, but no interaction was detected when the non-transgenic rice was used as a negative control (**Figure 3A**).

Quantification of the mAb was performed by comparing the OD450 reading of each sample to a standard curve produced by a commercial Bevacizumab (Avastin) standard (**Figure 3B**). The Bevacizumab contents in four selected transgenic lines were from 160.7 to 242.8 mg/Kg FW (**Figure 3C**).

### Extraction and Purification of mAb Expressed in Transgenic Rice Callus

**FIGURE 4:**
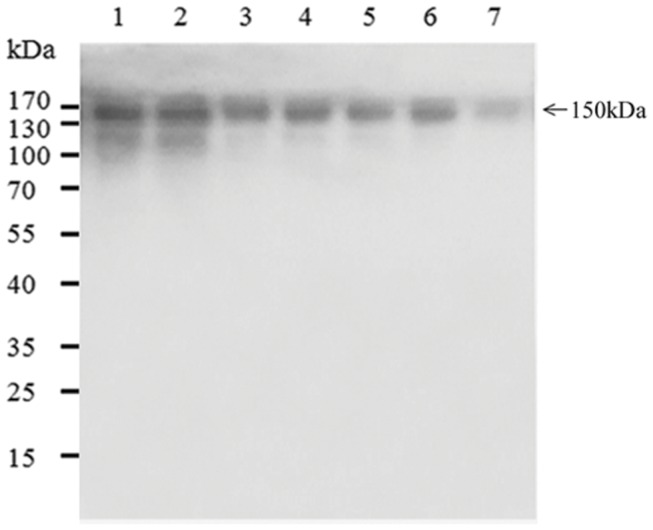
**SDS-PAGE of purified Bevacizumab mAb in transgenic rice.** 5 μg (lane 1), 2 μg (lane 3), and 1 μg (lane 5) mAbs purified from pUNBevaHL transformed rice callus, 5 μg (lane 2), 2 μg (lane 4), and 1 μg (lane 6) mAbs purified from pUNBevaHL-KDEL transformed rice callus, and 0.5 μg commercial Bevacizumab mAb (lane 7) were analyzed by non-reducing SDS-PAGE and stained with Coomassie blue. The major 150 kDa mAb band was indicated.

MAbs from two transgenic lines (UNBevaHL-3-15 and UNBevaHL-KDEL-5) were purified by Protein A affinity chromatography from 50 g of callus material from each transgenic line. 135.1 and 101.1 mg/Kg FW mAbs were recovered from the two transgenic lines, corresponding to a recovery rate of 55.6 and 59.8%, respectively (**Table 1**). The purities of the mAbs were detected by running purified samples on a SDS-PAGE, which showed mainly the ∼150 kDa mAb band. However, weak lower molecular weight bands were observed when 5 μg proteins (lanes 1 and 2) were loaded (**Figure 4**).

**Table 1:** Recovery of Bevacizumab mAb expressed in transgenic rice callus.

	UNBevaHL-3-15	UNBevaHL-KDEL-5
Fresh callus weight (g)	50	50
mAb yield (mg/Kg FW)	135.1	101.1
mAb content in callus (mg/Kg FW)	242.8	169.1
Recovery rate (%)	55.6	59.8


### Affinity Determination of Rice-derived Bevacizumab by ELISA

**FIGURE 5:**
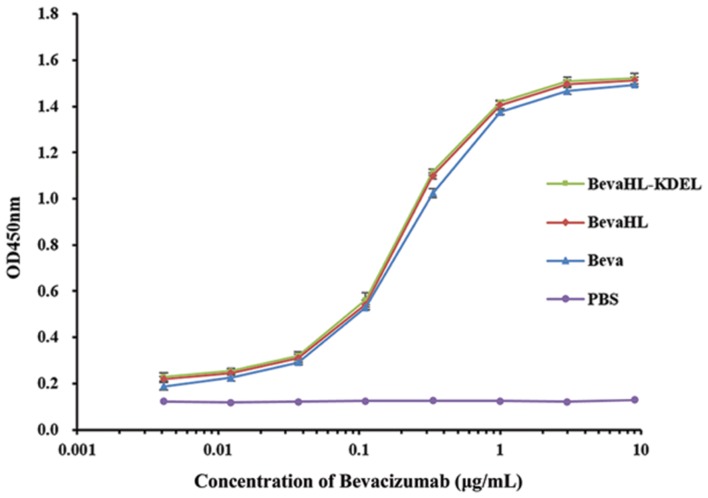
**Bevacizumab binding activity to VEGF measured by ELISA.** Purified Bevacizumab mAbs from transgenic rice callus with the pBevaHL-KDEL construct (green square) and the pBevaHL construct (red diamond), a commercial Bevacizumab (blue triangle, positive control), and 1× PBS buffer without Bevacizumab (purple dot, negative control) were analyzed by ELISA for binding activities to hVEGF. Each measurement was performed in triplicates.

To compare the binding affinity of the rice-derived mAbs with the commercial Bevacizumab to VEGF, an ELISA was performed with the two mAbs with and without the KDEL ER retention signal purified from rice callus (**Table 1**) and a commercial Bevacizumab control. As shown in **Figure 5**, there was no significant difference in binding affinity between the two Bevacizumab produced from rice callus with or without the KDEL signal peptide, and no significant difference was detected between the rice-derived mAbs to the commercial Bevacizumab.

### N-glycan Analysis of Rice-derived Bevacizumab

Tryptic digested glycopeptides from two rice-derived Bevacizumab mAbs differing in the KDEL ER retention signal peptide were analyzed by liquid chromatography tandem mass spectrometry (**Table 2**; **Supplementary Figure S3**). Both mAbs had high percentages of plant-specific glycosylation with β1, 2-xylose and α1, 3-fucose. The total percentages of plant-specific glycosylation including MMXF (M3Gn2X1F1), GnMXF (M3Gn3X1F1), and GnGnXF (M3Gn4X1F1) were 56.7% for mAb without the KDEL peptide and 46.8% for that with KDEL. Other forms of glycosylation including high mannose types, Man3 (M3Gn2), Man4 (M4Gn2), Man5 (M5Gn2), Man6 (M6Gn2), Man7 (M7Gn2), Man8 (M8Gn2), and Man9 (M9Gn2), paucimannosidic glycans as well as non-glycosylation were also observed in both mAbs produced in transgenic rice callus.

**Table 2:** Mass spectrometric analysis of glycopeptides from purified mAb after tryptic digestion.

Glycoform	Relative abundance (%)	
	
	pUNBevaHL	pUNBevaHL-KDEL
Non-glycosylated	2.7	10.8
Single GlcNAc (Gn)	16.3	26.1
Man3 (M3Gn2)	9.4	1.6
Man4 (M4Gn)	1.9	1.2
Man5 (M5Gn2)	2.3	1.9
Man6 (M6Gn2)	1.7	1.6
Man7 (M7Gn2	2.6	2.4
Man8 (M8Gn2)	4.0	5.7
Man9 (M9Gn2)	2.4	1.9
MMXF (M3Gn2X1F1)	19.4	14.6
GnMXF (M3Gn3X1F1)	7.6	5.1
GnGnXF (M3Gn4X1F1)	29.7	27.1


*GlcNAc, *N*-acetylglucosamine (Gn); F, fucose; Man, mannose (M); X, xylose.*

## Discussion

In this report, we designed and expressed a biologically functional mAb (Bevacizumab) in transgenic rice callus. The plant expression system has many advantages including high-level productivity, easy management and scaling-up, safety and low cost. To maximize the benefit of the plant expression system, many strategies have been developed to improve the expression and to increase the production. For example, transgenes can be stably integrated into the nuclear genome or transiently expressed using viral or Agrobacterium vectors (Vaquero et al., 1999; Kathuria et al., 2002; Rodriguez et al., 2005; Sainsbury et al., 2008; Villani et al., 2009). In addition, stable expression in cell cultures can produce uniform recombinant proteins in controlled environments by using bioreactors so that good manufacturing practice (GMP) can be applied (Shih and Doran, 2009; Xu et al., 2011; Huang and McDonald, 2012). In this study, we produced bioactive Bevacizumab in stably transformed rice callus and the rice-derived Bevacizumab content reached 242.8 mg/Kg FW (**Figure 4**). With the development of the rice suspension culture system, it is possible to scale up the production to reach large-scale production by using the bioreactor technology such as the continuous or semi-continuous production system to reduce the production time and manufacturing cost (Tekoah et al., 2015; Yang et al., 2015; Corbin et al., 2016).

The complete mAb consists of two identical LCs and HCs connected by disulfide bonds. Low-level expression of mAbs in previous reports was mostly affected by the expression strategies. Unbalanced expression of the LC and HC was unavoidable in the previous systems when the two genes coding for the LC and HC were expressed separately, or expressed in one bicistronic mRNA separately by the IRES because the downstream open reading frame (ORF) was only expressed at ∼10% level of the upstream one (Minskaia and Ryan, 2013; Minskaia et al., 2013). Unbalanced expression of LC and HC is unfavorable to the yield and quality of mAb production because efficient assembly and modification of mAb seem to require an optimal ratio of LC and HC (Schlatter et al., 2005; Ho et al., 2013b; Chng et al., 2015). However, contradictory results were reported on the optimal ratio of LC and HC in recombinant mAb production. Excessive expression of LC was considered to be beneficial for mAb production because excess LC would allow more efficient mAb folding, assembly, and the clearance of HC, which is prone to aggregation when expressed in excess (Vanhove et al., 2001; Lee et al., 2009). Ho et al. (2013b) reported higher titers of mAb production at a LC:HC ratio of 3.43. Similarly, Schlatter et al. (2005) observed efficient production of three mAbs with LC:HC peptide ratios of 2, 2.5, and 3.1 in CHO cells. On the contrary, separate groups also reported that excessive HC was beneficial to IgG expression (Jiang et al., 2006; Fallot et al., 2009). However, excessive expression of either LC or HC produced LC or HC dimmer and monomer molecules besides the LC_2_HC_2_ mAb (Ho et al., 2013b). Unlike the other systems, using of 2A self-cleavage peptide can ensure equal molars expression of LC and HC peptides to avoid unbalanced expression, and thus produce high levels of mAbs (Chng et al., 2015).

The FMDV RNA genome contains a single ORF, which encodes a polyprotein. The FMDV 2A peptide between the 2A and 2B regions of the polyprotein is self-cleaved to produce the viral 2A and 2B proteins (Ryan et al., 1991). The self-cleavage of 2A peptide is co-translational by a “Stop-Go” or “Stop-carry on” recoding mechanism, by which the upstream protein translation is paused by the 2A sequence and the peptide backbone is broken between the last two amino acids, Glycine (G) and Proline (P) of the 2A peptide (Atkins et al., 2007; Doronina et al., 2008; Sharma et al., 2011). The 2A peptide also drives a translational recoding of the downstream protein. Unlike other systems, 2A peptide-mediated cleavage is not dependent on cellular factors in different cell types because the self-cleavage activity is only dependent on the ribosomes, which are structurally conserved among all eukaryotic organisms. Thus, the 2A peptide is active in all eukaryotic systems, and has been used widely as a co-expression tool in the expression of two or more proteins (Donnelly et al., 2001; de Felipe et al., 2006). Recently, 2A peptide was applied in stable and high levels expression of mAbs in CHO cells and mice, and was considered to have potential applications of *in vivo* delivery of mAbs in gene therapy (Fang et al., 2005; Chng et al., 2015). 2A peptide was also applied in the production of a single chain antibody in plants (Smolenska et al., 1998). However, the application of 2A in the production of complete mAb in plants has not been reported so far.

We reported the expression of a mAb (Bevacizumab) LC and HC as a polyprotein with the 2A linker from FMDV in one gene expression cassette driven by a strong constitutive ubiquitin promoter in transgenic rice callus. High levels of functional antibodies were produced (**Figure 3**). We achieved the expression of more than 200 mg/Kg FW of Bevacizumab mAb in transgenic rice cells, which was higher than most of the reported recombinant mAb expression systems (Gomord et al., 2004; Girard et al., 2006; Villani et al., 2009; De Muynck et al., 2010; Madeira et al., 2015). The high levels of expression could be attributed to the following factors. First, the codon optimization makes the mRNA stable and the protein translation efficient. Second, a strong constitutive ubiquitin promoter increases the transgene transcription level. Third, single copy transgene by the *Agrobacterium*-mediated transformation reduces the chances of gene silencing. Last but not least, the expression of a polyprotein using 2A self-processing peptide favors the accumulation of functional mAb. The using 2A peptide expression system may affect the mAb yield by the following ways. First, the self-cleavage of 2A linked polyprotein produces equal molecules of HC and LC, which might be an optimal ratio for mAb accumulation. Western blot revealed that the LC and HC of Bevacizumab expressed with 2A polypeptide were at similar levels in most transgenic lines, but unbalanced expressions of LC and HC were observed when they were on different expression cassettes (**Figure 3**). Although the excess of either LC or HC might speed up the mAb assembly as shown in a mathematical modeling (Gonzalez et al., 2002), a 1:1 LC:HC ratio is more economical because IgG mAbs have equal numbers of LC and HC subunits. Second, the self-cleavage is co-translational, so that both the LC and HC are simultaneously produced and mobilized into ER, where mAb can be immediately assembled and folded. Third, correct and complete assembly of mAb may reduce proteolytic degradation of either LC or HC monomers or dimmers when present in excess because incorrectly or incompletely folded antibodies are susceptible to protease attack (Gardner et al., 1993). Post-translational protein degradation has been a common phenomenon that significantly influences the yield of foreign protein production (Doran, 2006; De Muynck et al., 2010; Desai et al., 2010; Xu et al., 2011; Hehle et al., 2016). But, the degradation of mAb was not observed in our studies based on the Western blot results. This is probably because of the correct and complete assembly of mAb from LC and HC subunits generated from the 2A self-cleavage polyprotein (**Figure 2C**). The weak lower molecular weight bands observed in **Figure 4** were probably degradation products during the process of protein extraction and purification because such bands were not observed before this process (**Figure 2C**).

Glycosylation is an important property of recombinant mAb that affects its binding affinity and circular half-life, and causes immunogenicity (Saint-Jore-Dupas et al., 2007; Reusch and Tejada, 2015). It has been shown that plant expressed proteins are differently modified as compared to mammalian systems. The plant-specific glycosylation including the β 1, 2-xylose and a core α 1, 3-fucose takes place in the Golgi apparatus. To avoid plant-specific glycosylation, two strategies are generally used: to keep the recombinant protein in the ER with an ER retention signal peptide or to knockout plant genes coding the plant-specific glycosylation enzymes by glycoengineering (Strasser et al., 2008, 2014; Castilho and Steinkellner, 2012; Castilho et al., 2014; Kurogochi et al., 2015). In this report, we tried to target both the HC and LC to ER by adding KDEL signal peptides at the C-termini, but were unsuccessful in preventing the plant-specific glycosylation (**Table 2**), indicating that the KDEL signals were not functioning properly. One reason for this malfunction of the KDEL signal could be related to results of the self-processing of the 2A peptide linker between the HC and LC. It was reported that the cleavage of the 2A peptide between the last two amino acids, Glycine (G) and Proline (P) would result in the adding of a GSGQLLNFDLLKLAGDVESNPG peptide to the C-terminal of the upstream protein and a P amino acid residue to the N-terminal of the downstream one (Chng et al., 2015). In our design (**Figure 1**), the adding of the 2A peptide to the C-terminal of the HC would abolish the KDEL signal, which only functions when present at the C-terminal of a protein. The loss of KDEL signal in upstream protein can be solved by adding a furin protease recognition site upstream of 2A, which will remove the residues of the 2A peptide (Chng et al., 2015). Although this strategy can solve the ER retention problem in future studies, the retention of recombinant mAb in ER with the KDEL signal will limit the protein secretion and invariably increase the downstream process cost. Thus, an alternative approach by glycoengineering to silence or knockout the plant-specific glycosylation genes and to introduce mammalian glycosylation genes into plants could be a better solution (Strasser et al., 2008, 2014; Castilho and Steinkellner, 2012).

Glycosylation affects the antibody-dependent cell-mediated cytotoxicity (ADCC) of recombinant mAbs. For example, fucose-free mAb (13F6) produced in glycoengineered tobaccos was demonstrated to have higher binding affinity to Fc_γ_R and enhanced potency against Ebola virus (Zeitlin et al., 2011). However, Bevacizumab functions through the binding and neutralization of secreted VEGF, but lacks the ADCC (Wang et al., 2004). The biological activity of rice-derived Bevacizumab mAb (including KDEL tagged and non-tagged) was analyzed by an ELISA-based binding assay to its hVEGF antigen (**Figure 5**). No significant difference was observed between the two rice expressed mAbs and between the rice expressed mAb and the commercial Bevacizumab control, indicating that the rice expressed Bevacizumab mAb is functional. It remains unknown if the rice expressed Bevacizumab mAb have other comparable properties such as the neutralization of hVEGF *in vivo*, the circular half-life in clinical studies, and most importantly the adverse immunogenicity related to plant expressed recombinant proteins, which need to be characterized in future studies.

In conclusion, we reported an efficient recombinant mAb expression system in transgenic rice callus using the FMDV 2A self-cleavage peptide. The cleavage of 2A peptide linked polyprotein in one gene expression cassette produced equal molecules of HC and LC of a mAb, allowed efficient assembly and folding of functional antibody, and produced recombinant mAb at high levels.

## Author Contributions

WY and DL conceived, designed, and supervised the study, discussed the results, and wrote the manuscript. LC and XY performed the experiments, analyzed the data, and wrote the manuscript. All authors approved the final manuscript.

## Conflict of Interest Statement

The authors declare that the research was conducted in the absence of any commercial or financial relationships that could be construed as a potential conflict of interest.

## Footnotes

1 http://www.avastin.com/patient

2 http://www.drugbank.ca/drugs/DB00112

3 http://genomes.urv.es/OPTIMIZER/

4 http://www.kazusa.or.jp/codon/cgi-bin/showcodon.cgi?species=311553

5 https://imagej.nih.gov/ij/index.html

6 http://www.expasy.org/tools/peptide-mass.html
