# Supplementary material for: Efficient Production of a Bioactive Bevacizumab Monoclonal Antibody Using the 2A Self-cleavage Peptide in Transgenic Rice Callus
Source: Front Plant Sci. 2016 Aug 9;7:1156. doi: 10.3389/fpls.2016.01156 (PMC4977302; doi:10.3389/fpls.2016.01156)
Supplement: Supplementary file 1 [file Presentation_1.PDF]

Supplementary data

Supplementary Method S1 Sothern blot analysis of transgenic rice lines.

Genomic DNAs from calli of wild type and selected transgenic rice lines, BevaHL-1, -2,-3, -4,-7, -3-910, -3-15, BevaHL-KDEL-1, -2, -5, -6, -8, -10, -12 were isolated by SDS method with modifications (Edwards K, Johnstone C and Thompson C [1991] A simple and rapid method for the preparation of plant genomic DNA for PCR analysis. Nucleic Acids Res 19:1349). 0.1 g sample was ground to a fine powder in liquid nitrogen by a mortar and a pestle. The powder was transferred to a 2 mL Eppendorf tube and 400 μL DNA extraction buffer (0.5 M NaCl, 0.1 M Tris-HCl, 0.05 M EDTA and 0.6% (w/v) SDS, pH 8.0) was added to mix by vortexing for 1 min. The sample was left at room temperature for 10 min. After 10 min centrifugation at 14,000 rpm at room temperature, 300 μL supernatant was transferred to a new 1.5 mL tube. To precipitate the genomic DNA, the sample was mixed with 1 volume of isopropanol and kept at -20 ℃ for 10 min. Then, the sample was centrifuged at 5,000 rpm for 10 min at room temperature and the resulted DNA pellet was re-suspended in 0.6 mL of 1 M NaCl. The DNA solution was then mixed with 1 volume of chloroform, and centrifuged at 12,000 rpm for 10 min. DNA in the supernatant was precipitated again in a new 1.5 mL tube. DNA pellet was washed with 500 μL of 70% (v/v) ethanol, air-dried and then dissolved in 50 μL sterilized Milli-Q water containing 10 units RNase A for 5 min at room temperature. The isolated genomic DNAs were qualified by a NanoDrop Lite Spectrophotometer (Thermo Fisher Scientific Inc., Wilmington, Delaware, USA) and checked by gel electrophoresis.

Twenty micrograms of genomic DNA per sample were digested with 150 units of *Eco*R I restriction enzyme at 37 ℃ overnight, fractioned in 1.0 % (w/v) agarose gel at 60 volts for 4 h, denatured and transferred to Hybond N^+ membranes. Digoxygenin (DIG) labeled Bevacizumab full-length LC DNA fragment and full-length *Hpt*II gene were synthesized by PCR and used as probes. Prehybridization, washing, and chemiluminescent detection of the blot were performed according to the manufacturer’s instructions (Roche Diagnostics GmbH, Germany). The primers used for amplifications of the Bevacizumab

LC and *hpt*II gene fragments were:

BLC forward 5′-ATGAAGTACCTCCTCCCTACCGCGG-3′;

BLC reverse 5′-TCAGAGTTCGTCCTTGCATTCGCC-3′;

*Hpt*II forward 5’-ATGAAAAAGCCTGAACTCACCGCG-3’;

*Hpt*II reverse 5’-CTATTTCTTTGCCCTCGGACGAGTGC-3’.

Supplementary Figure S1 Southern blot analysis of transgenic rice lines.

Lane1: a pUNBevaHL-KDEL plasmid control, lane 2: a nontransgenic rice negative control, lane 3-9: pUNBevaHL transformed transgenic plants, lane 10-16: pUNBevaHL-KDEL transgenic plants. DNA fragment sizes were indicated at the left side. Both the pUNBevaHL and pUNBevaHL-KDEL constructs contain two *Eco*R I digestion sites flanking the ~3.0 kb *BHC* and *BLC* polyprotein gene. Transgenic plants with intact *BHC* and *BLC* polyprotein coding region are revealed by ~3.0 kb hybridization with the *BLC* probe (a). The transgene copy numbers are revealed by hybridization with the *HptII* gene probe because there is only one *Eco*R I digestion site in the T-DNA regions of the constructs (b).

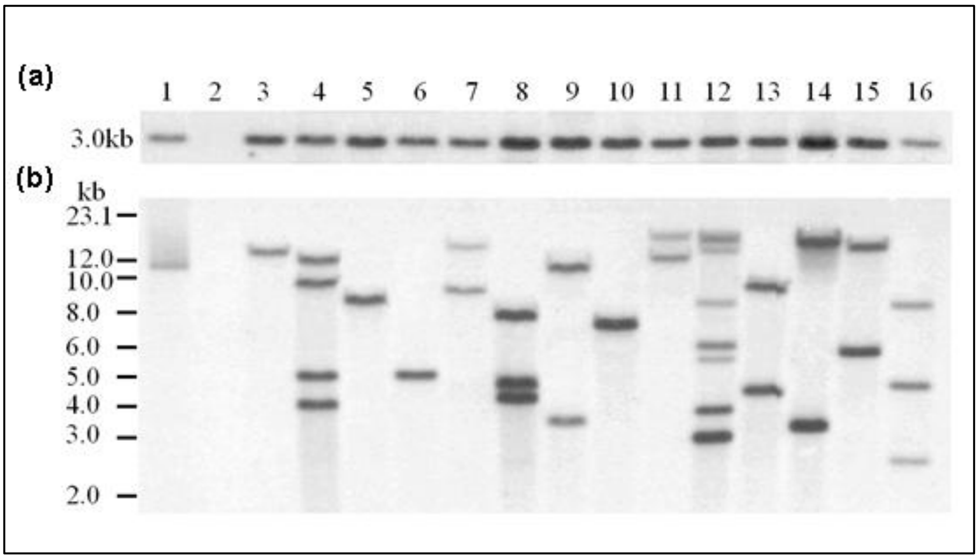

Supplementary Figure S2 Transformation and regeneration of rice with pUNBevaHL and pUNBevaHL-KEDL. (a) Resistant calli on selection media; (b) transgenic rice regenerated in culture bottles; (c) T0 transgenic plants. Bar=10cm.

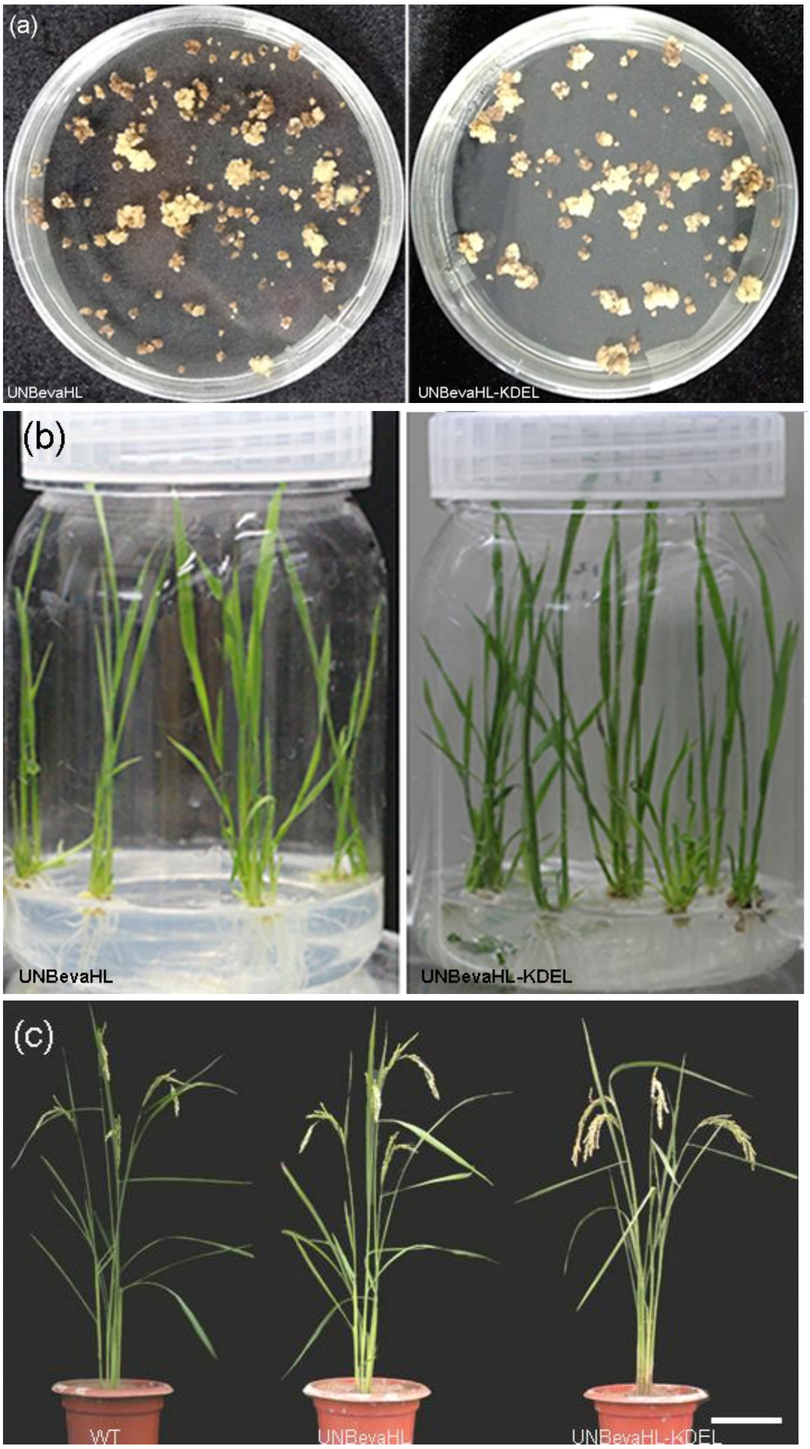

Supplementary Figure S3 Deconvoluted liquid chromatography-mass spectra of glycosylated peptides containing asparagine-303(Asn-303) from the Bevacizumab Fc region. Glycopeptides were derived from the peptic digestion of the pUNBevaHL (a) and pUNBevaHL-KDEL (b) Bevacizumab heavy chain (HC).

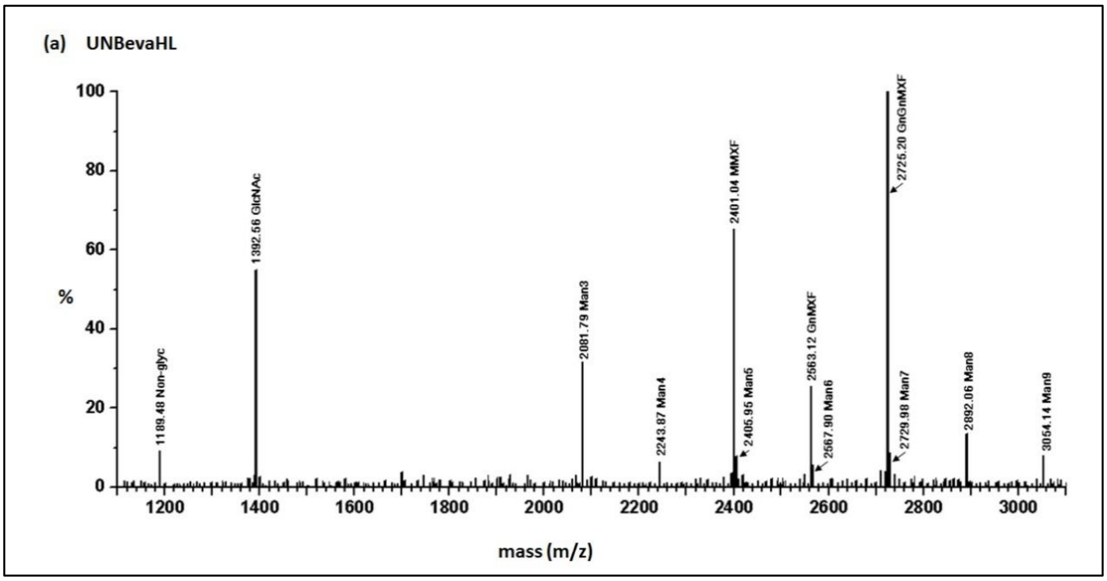

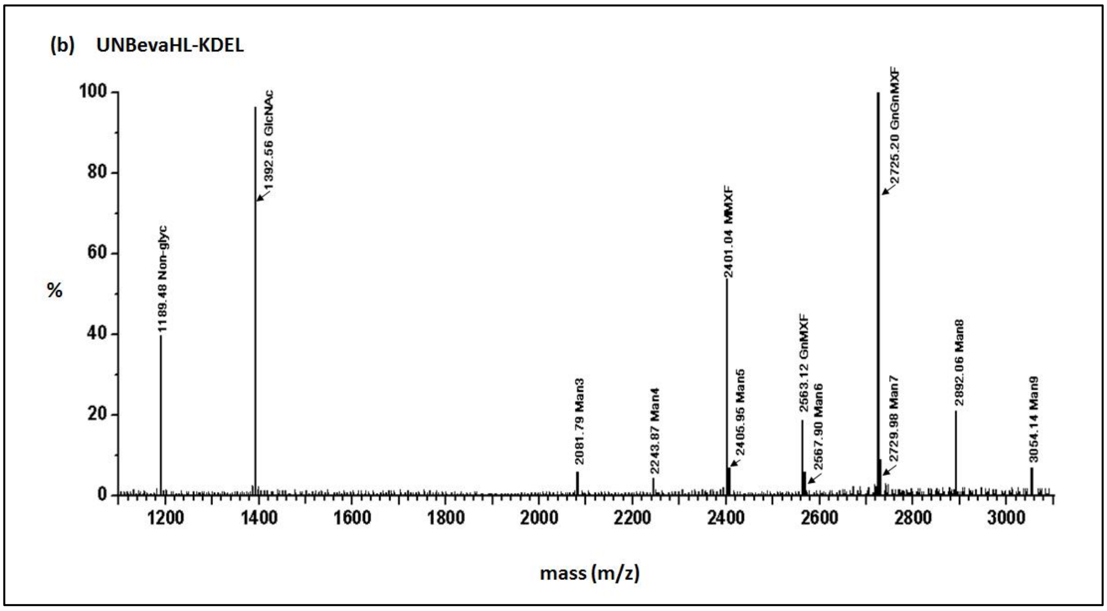

Supplementary Table S1 LC:HC ratio of expressed Bevacizumab in transgenic lines

Sample	LC:HC ratio
Bevacizumab control	
PC	0.99
Transgenic lines with two gene expression cassettes	
UNBHLC-1	0.95
UNBHLC-2	3.07
UNBHLC-3	2.54
UNBHLC-4	1.36
UNBHLC-5	19.23
UNBHLC-6	3.77
UNBHLC-7	2.47
UNBHLC-8	1.17
Average ± SD	4.32 ± 6.10
Transgenic lines expressing a polyprotein with a 2A self-cleavage peptide	
UNBevaHL-1	0.68
UNBevaHL-2	1.18
UNBevaHL-3	1.00
UNBevaHL-4	0.99
UNBevaHL-5	1.00
UNBevaHL-KDEL-1	0.11
UNBevaHL-KDEL-2	0.60
UNBevaHL-KDEL-3	1.49
UNBevaHL-KDEL-4	1.90
Average	0.99 ± 0.52

Signal densities of LC and HC bands in western blot (Figure 2a and 2b) were analyzed by using the ImageJ program (https://imagej.nih.gov/ij/index.html). The LC:HC ratio was calculated by dividing the density of LC by that of the HC in each sample. A commercial Bevacizumab (PC) was used as a control. SD means standard deviation.
